# Efficacy of robot-assisted partial nephrectomy compared to conventional laparoscopic partial nephrectomy for completely endophytic renal tumor: a multicenter, prospective study

**DOI:** 10.1007/s10147-024-02599-9

**Published:** 2024-08-07

**Authors:** Nobuyuki Hinata, Sae Murakami, Yuzo Nakano, Isao Hara, Tsunenori Kondo, Shuzo Hamamoto, Ryoichi Shiroki, Jun Nagayama, Mutsushi Kawakita, Masatoshi Eto, Osamu Ukimura, Atsushi Takenaka, Toshio Takagi, Masaki Shimbo, Haruhito Azuma, Tetsuya Yoshida, Junya Furukawa, Naoki Kawamorita, Masato Fujisawa

**Affiliations:** 1 Department of Urology, Graduate School of Biomedical and Health Sciences Hiroshima University, 1-2-3, Kasumi Minami-ku, Hiroshima, 734-8551 Japan; 2 Department of Urology, Kobe University Graduate School of Medicine, Kusunoki-cho, Chuo-ku, Kobe, Hyogo 657-0017 Japan; 3 Clinical and Translational Research Center, Kobe University Hospital, Kusunoki-cho, Chuo-ku, Kobe, Hyogo 650-0017 Japan; 4 Department of Urology, Wakayama Medical University, 811-1 Kimiidera, Wakayama, Wakayama 641-8509 Japan; 5 Department of Urology, Tokyo Women’s Medical University, Adachi Medical Center, 4-33-1, Kohoku, Adachi-Ku, Tokyo, 123-8558 Japan; 6 Department of Nephro-urology, Nagoya City University, 1, Kawasumi Mizuho-cho, Mizuho-ku, Nagoya, Aichi 467-8602 Japan; 7 Department of Urology, Fujita-Health University School of Medicine, Toyoake City, 1-98 Dengakugakubo, Kutsukake-cho, Toyoake, Aichi Japan; 8 Department of Urology, Nagoya University Graduate School of Medicine, 65 Tsurumai-cho, Showa-ku, Nagoya, Aich 466-8560 Japan; 9 Department of Urology, Kobe City Medical Center General Hospital, 2-1-1 Minatojima Minamimachi, Chuo-ku, Kobe, Hyogo 650-0047 Japan; 10 Department of Urology, Kyushu University, 744 Motooka Nishi-ku, Fukuoka, Fukuoka 819-0395 Japan; 11 Department of Urology, Graduate School of Medical Science, Kyoto Prefectural University of Medicine, 465, Kajiicho, Kawaramachi-Hirokoji, Kamigyo-ku, Kyoto, Kyoto 602-8566 Japan; 12 Division of Urology, Department of Surgery, Graduate School of Medicine, Faculty of Medicine, Tottori University, 36-1, Nishicho, Yonago, Tottori 683-8504 Japan; 13 Department of Urology, Tokyo Women’s Medical University Hospital, 8-1, Kawadacho, Shinjuku-Ku, Tokyo, 162-8666 Japan; 14 Department of Urology, St. Luke’s International Hospital, 9-1, Akashicho, Chuo-Ku, Tokyo, 104-0044 Japan; 15 Department of Urology, Osaka Medical and Pharmaceutical University Hospital, 2-7 Daigakumachi, Takatsuki, Osaka 569-8686 Japan; 16 Department of Urology, Shiga University of Medical Science Hospital, Seta-Tsukinowatyo Seta, Ohtsu, Shiga 520-2192 Japan; 17 Department of Urology, Tokushima University Graduate School of Biomedical Sciences, 3-18-15, Kuramoto-cho, Tokushima, Tokushima 770-8503 Japan; 18 Department of Urology, Tohoku University Graduate School of Medicine, Seiryo-machi, Aoba-ku, Sendai, Miyagi 980-8574 Japan

**Keywords:** Nephrectomy, Robotic surgical procedures, Carcinoma, Renal cell, Renal insufficiency

## Abstract

**Background:**

This study aimed to compare the efficacy of robot-assisted partial nephrectomy for completely endophytic renal tumors with the reported outcomes of conventional laparoscopic partial nephrectomy and investigate the transition of renal function after robot-assisted partial nephrectomy.

**Methods:**

We conducted a prospective, multicenter, single-arm, open-label trial across 17 academic centers in Japan. Patients with endophytic renal tumors classified as cT1, cN0, cM0 were included and underwent robot-assisted partial nephrectomy. We defined two primary outcomes to assess functional and oncological aspects of the procedure, which were represented by the warm ischemic time and positive surgical margin, respectively. Comparisons were made using control values previously reported in laparoscopic partial nephrectomy studies. In the historical control group, the warm ischemia time was 25.2, and the positive surgical margin was 13%.

**Results:**

Our per-protocol analysis included 98 participants. The mean warm ischemic time was 20.3 min (99% confidence interval 18.3–22.3; p < 0.0001 vs. 25.2). None of the 98 participants had a positive surgical margin (99% confidence interval 0–5.3%; p < 0.0001 vs. 13.0%). The renal function ratio of eGFR before and after protocol treatment multiplied by splits was 0.70 (95% confidence interval: 0.66–0.75). Factors such as preoperative eGFR, resected weight, and warm ischemic time influenced the functional loss of the partially nephrectomized kidney after robot-assisted partial nephrectomy.

**Conclusions:**

Robot-assisted partial nephrectomy for completely endophytic renal tumors offers a shorter warm ischemia time and comparable positive surgical margin rate compared with conventional laparoscopic partial nephrectomy.

**Supplementary Information:**

The online version contains supplementary material available at 10.1007/s10147-024-02599-9.

## Introduction

Preserving renal function while achieving oncological clearance is a unique surgical challenge in renal cell carcinoma. Partial nephrectomy has become the standard of care for small renal masses amenable to nephron-sparing strategies [1]. The surgical complexity of these procedures varies, particularly with renal hilar and completely endophytic tumors. These are considered among the most challenging because of their anatomical positions, as categorized by the R.E.N.A.L. [2] or PADUA [3] scoring systems. The debate within the surgical community primarily centers on achieving a balance between oncological efficacy and patient safety. Oncological efficacy is typically gaged by the completeness of tumor resection, as evidenced by surgical margins. Additionally, patient safety has a dual focus: minimizing perioperative adverse events and preserving long-term renal function. The latter is of particular importance in partial nephrectomy, where nephron conservation is a critical goal. Warm ischemia time (WIT) as well as remnant healthy renal parenchyma are reported to be surgery-related factors related to long-term postoperative renal function [4, 5].

The advantage of robot-assisted partial nephrectomy (RAPN) over conventional laparoscopic partial nephrectomy (cLPN) has been reported [6–8]. Moreover, the advent of RAPN has introduced potential advantages for the management of complex renal tumors, such as hilar tumors [9, 10] or completely endophytic tumors. The key benefit of RAPN lies in the multijoint movement of its robotic arms, which offers enhanced precision, superior maneuverability, and improved access to difficult-to-reach tumors.

Our study was a single-arm trial, where outcomes from RAPN were compared with historical cLPN data while focusing on oncological and renal functional outcomes. We particularly emphasized the importance of the WIT as a surrogate marker of postoperative renal function. Our research aimed to provide a deeper understanding of the oncological and functional outcomes in the context of modern renal cancer surgery. We also investigated the key factors affecting renal function at POD 180, providing insight into the medium-term impacts of RAPN.

## Patients and methods

### Overview and settings

Our study was conducted as a prospective, multicenter, single-arm, open-label clinical trial over a 17-month recruitment period (trial registration number: jRCT1052200016). We compared our findings with those of previous studies on cLPN. This investigation included 17 distinguished academic hospitals across Japan equipped with da Vinci surgical systems (Intuitive Surgical, Inc. (Sunnyvale, CA, USA)). This study was approved by a central review board (approval number C190037), and all participants gave their written informed consent for participation. Although this study is planned for a 5-year follow-up period to evaluate long-term outcomes, this report includes findings up to POD180 to present short and midterm results.

### Exclusions

Eligible patients were those scheduled for partial nephrectomy because of completely endophytic renal tumors classified as cT1, cN0c, M0. A completely endophytic renal tumor is defined as one that does not protrude from the renal cortex. Patients with a history of synchronous cancer and kidney transplant recipients were excluded.

### Procedure

The surgical technique adhered to the methodology detailed in prior research [10]. The approach for renal artery clamping was individualized by each participating institution and was not standardized for this study.

### Primary outcomes

The primary endpoints of this study were WIT and the proportion of positive surgical margins (PSM), emphasizing the importance of preserving renal function in urological surgery and achieving oncological efficacy through complete resection.

### Secondary outcomes

Secondary outcomes are as follows.

1. Achieving both negative tumor margins and WIT of ≤ 25 min.
2. Changes in the estimated glomerular filtration rate (eGFR).
3. Trifecta achievement, defined as follows: (a) negative surgical margins. b. Maintenance of ≥ 90% of the preoperative eGFR at POD 180. c. No pseudoaneurysms, postoperative bleeding, or urinary fistula (Clavien–Dindo ≥ 3) within POD 180.
4. Pre- and post-operative operated renal function variation, measured by split eGFR adjusted for renal scintigraphy-derived uptake ratios [11].
5. Time-free from the progression of chronic kidney disease (CKD). CKD progression was defined as worsening by one or more stages occurring after POD 30.

Secondary outcomes (2), (4), and (5) were geared toward evaluating renal function, while (1) and (3) were oriented toward both renal function and oncological outcomes.

### Sample size

To select historical data for comparison, we conducted a systematic search via PubMed in November 2019 using the search terms ["Laparoscopic Partial Nephrectomy" AND "complete endophytic renal tumors"] and ["Laparoscopic Partial Nephrectomy" AND "central tumor"]. We included original articles that reported on two or more patients. Four articles [12–15] met our inclusion critertia. Based on these four studies, the historical control for WIT was established at 25.2 min. For RAPN, the expected WIT was set at 22.2 min with a 7.9 standard deviation according to our internal data including previous report [10].

For sample size estimation, we adopted a mean WIT of 22 ± 7.9 min to test for superiority against the historical control. The study was designed with a stringent one-sided significance level of 0.5% (α = 0.005) and a power of 90% to account for multiple endpoint testing and reduce the risk of type I and II errors. These calculations determined a sample size of 95 subjects, which was increased to 100 to allow for any potential discontinuations or dropouts.

In the binomial test for PSM, utilizing the sample size calculated for WIT evaluation, with a 13% threshold (following previous studies [10]) and an anticipated PSM rate of 2%, at a one-sided significance level of 0.5%, the detection power exceeds 98%. The threshold was set at 13% following previous study. This suggests that the study is well-powered to assess this oncological endpoint.

### Statistical methods

Statistical analyses were performed to determine the efficacy and safety of the RAPN. For both primary endpoints, 95% and 99% confidence intervals (CIs) were estimated. A one-sample t-test was used to assess the significance of WIT reduction, with the alpha level set at 0.5% for one-sided tests. The PSM rate was analyzed using the binomial test against a threshold of 13%.

The mean estimates and their 95% CIs were computed for the remaining secondary endpoints. In analyzing factors contributing to the decline in renal function after surgery, the dependent variable was the percentage of post- and pre-operative split eGFR of the operated kidney. Independent factors included in the analysis were age, sex, baseline renal function, surgical complexity, surgical time, WIT, and weight of the resected kidney tissue.

Multivariate regression analyses were performed to identify predictors of split renal function, with each factor above entered into the model. If a factor has two or more variables, one of them is chosen in each model. Model selection was guided by the Akaike Information Criterion (AIC), with preference given to the model with the lowest AIC value. Exploratory analyses were performed using SAS 9.4 (SAS Institute, Cary, NC, USA).

## Results

### Patients

**Fig. 1:**
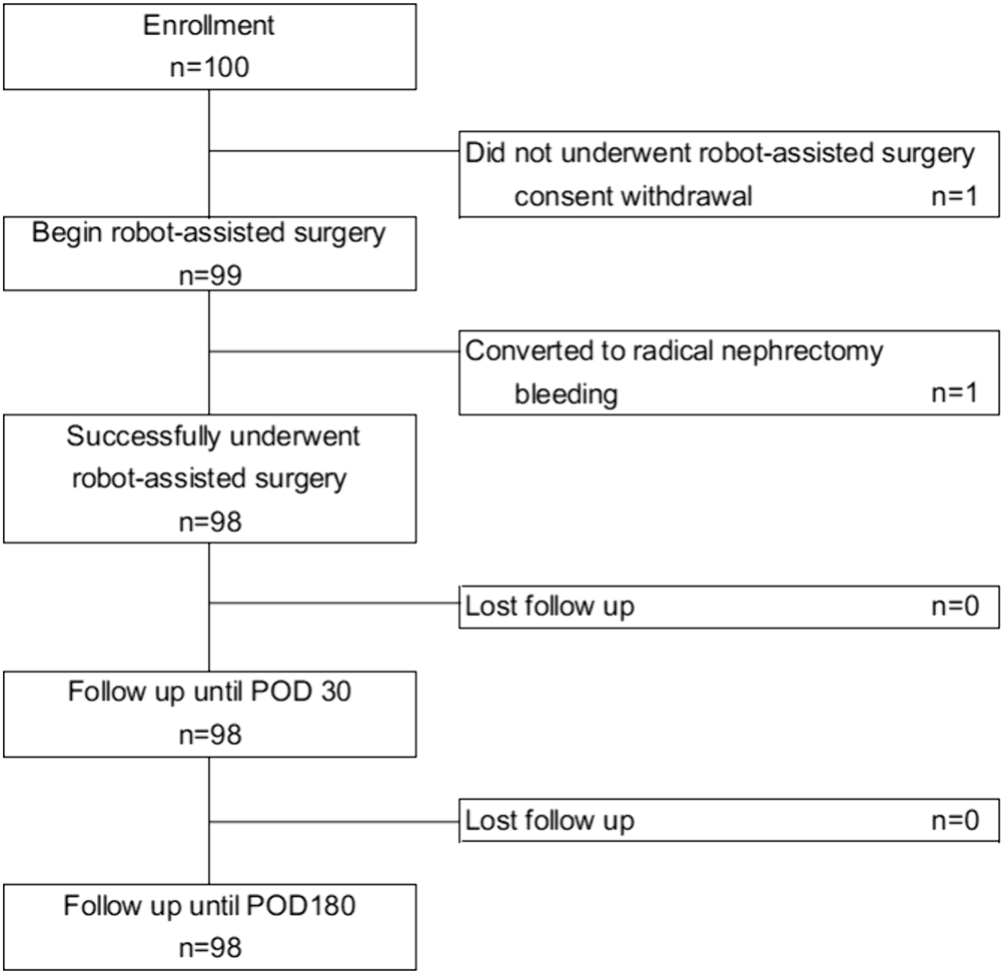
Trial flow chart

Of the 100 patients initially enrolled in the study, one withdrew consent before surgery. During surgery, one patient discontinued due to bleeding requiring conversion to radical nephrectomy. Consequently, 98 participants completed the intervention and were follow-up to POD 180 (Fig. 1). The detailed demographics and clinical characteristics of 98 patients are presented in Table 1. Regarding renal tumor complexity (which is assessed by the PADUA and R.E.N.A.L. scoring systems) nearly half of the patients were in the high complexity category. For a comparative overview of our findings with historical data, please refer to Supplementary Table S1.

**Table 1:** Characteristics of patients

Baseline characteristics	Median (range), n (%)
Patients (*n*)	98
Women, *n* (%)	37 (37.8)
Median age, years (range)	63 (31–87)
Median BMI, kg/m^2^ (range)	23.9 (16.4–33.1)
Median weight, kg (range)	64.3 (35.9–94.6)
*ASA–PS category, n(%)*	
1	42 (42.9)
2	48 (49.0)
3	6 (6.1)
NA	2 (2.0)
*Past operation*	
None	62 (63.3)
Laparoscopic surgery	5 (5.1)
Open surgery	31 (31.6)
Median serum creatinine, mg/dL (range)	0.79 (0.42–1.95)
Median eGFR, ml/min/1.73 (range)	69.1 (17.2–114.9)
Median eGFR (operative kidney), ml/min/1.73 (range)	36.0 (17.6–69.0)
*R.E.N.A.L. classification*	
Low complexity	6 (6.1%)
Moderate complexity	50 (51%)
High complexity	42 (42.9%)
Mean tumor diameter, cm (range)	2. 2 (0.8–5.5)
*Perioperative data*	
Median surgical time, min (range)	198 (105–340)
Median estimated blood loss, mL (range)	25 (1–64.3)
Median WIT, min (range)	19 (9–45)
*Perioperative complications*	
Total (*n*)	12
Clavien grade I/II (*n*)	9
Clavien grade III/IV (*n*)	3

*BMI* Body mass index, *ASA-PS* ASA physical status classification system, *eGFR* the estimated glomerular filtration rate, *WIT* warm ischemic time

### Primary outcomes

**Fig. 2:**
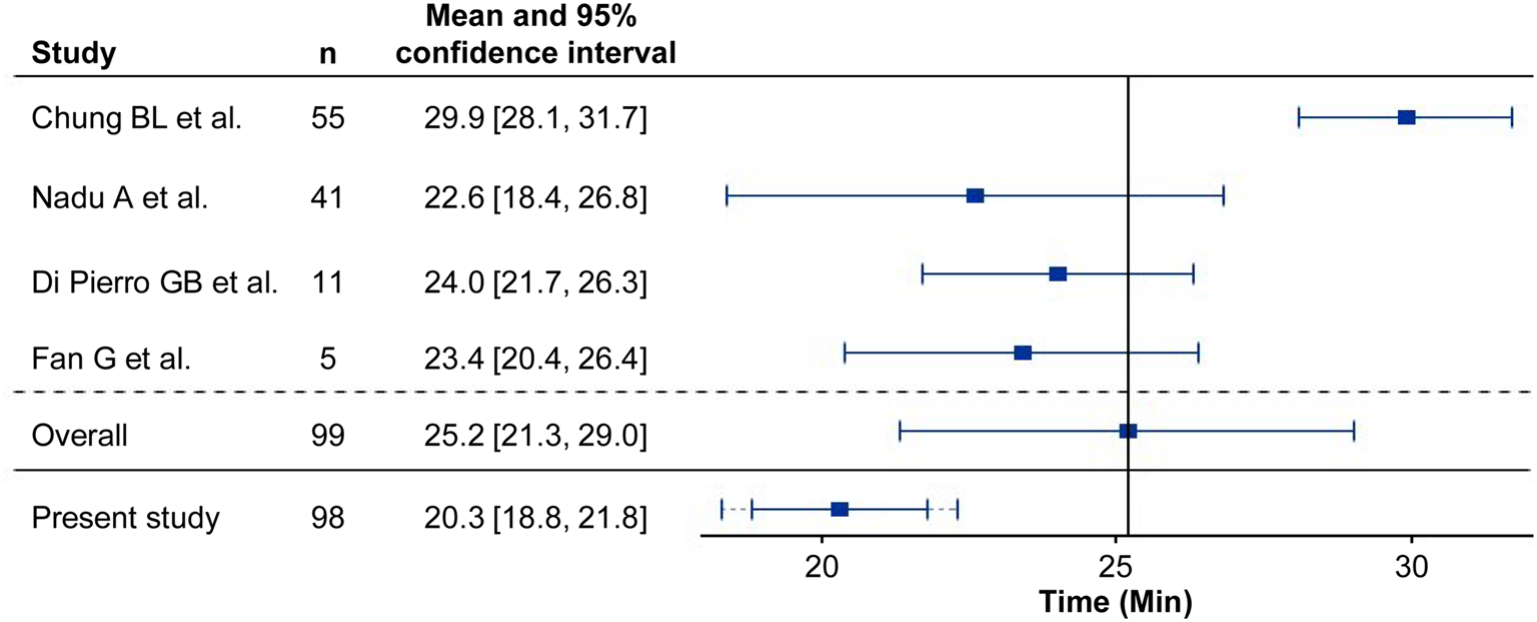
Mean WIT with the 95%CI of four historical studies and the present study. The dotted error bar is the 99%CI of the present study. A reference line at 25.2 min is included, representing the average WIT across the four historical studies. This line serves as a benchmark against which the WITs of the individual studies and our current trial data are compared

The mean WIT achieved in RAPN was 20.3 min, which was significantly less than the historical control value of 25.2 min (99% CI 18.3–22.3, p < 0.001, one-sided) (Fig. 2). Furthermore, the incidence of PSM was 0% in the cohort, markedly lower than the null hypothesis value of 13% (99% CI 0–5.3, p < 0.0001, one-sided). These findings collectively indicate that RAPN provided superior outcomes compared with cLPN data for these critical parameters.

### Secondary outcomes

#### Early outcomes

In the immediate postoperative period, we focused on the preservation of renal function and the oncological efficacy. Eighty (81.6%) of the patients achieved a WIT of 25 min alongside securing a negative surgical margin. Detailed results of each of these outcomes are presented in Table 2.

**Table 2:** Surgical outcome of robot-assisted surgery

	N	N/A	Yes n, (%)	(95%CI)
*Each primary outcome*				
WIT within 25 min	98	0	80, (81.6)	(72.5, 88.7)
Negative surgical margin	98	0	98, (100)	(96.3, 100)
Both above	98	0	80, (81.6)	(72.5, 88.7)
*Trifecta achievements*				
Achieve all of following Trifecta	98	0	47, (48.0)	(38.3, 57.7)
a. Negative surgical margin	98	0	98, (100)	(96.23, 100)
b. eGFR at postoperative day 180 is ≥ 90% of preoperative eGFR	98	1	47, (48.0)	(38.33, 57.5)
c. No significant complications* by 180 postoperative days	98	0	96, (98.0)	(92.9, 99.4)

*WIT* warm ischemic time

*Pseudoaneurysm, postoperative hemorrhage, urinary tract fistula of Clavien–Dindo classification grade 3 or higher. NA: not assessmented

#### Midterm outcomes

The midterm outcomes focused on renal function stability and the achievement of trifecta. The “trifecta” was achieved by 47 patients (48.0%, 95% CI 38.3–57.7) at POD 180. Details of the trifecta are shown in Table 2. Urinary leakage was observed in two patients, one of whom underwent nephrectomy.

**Fig. 3:**
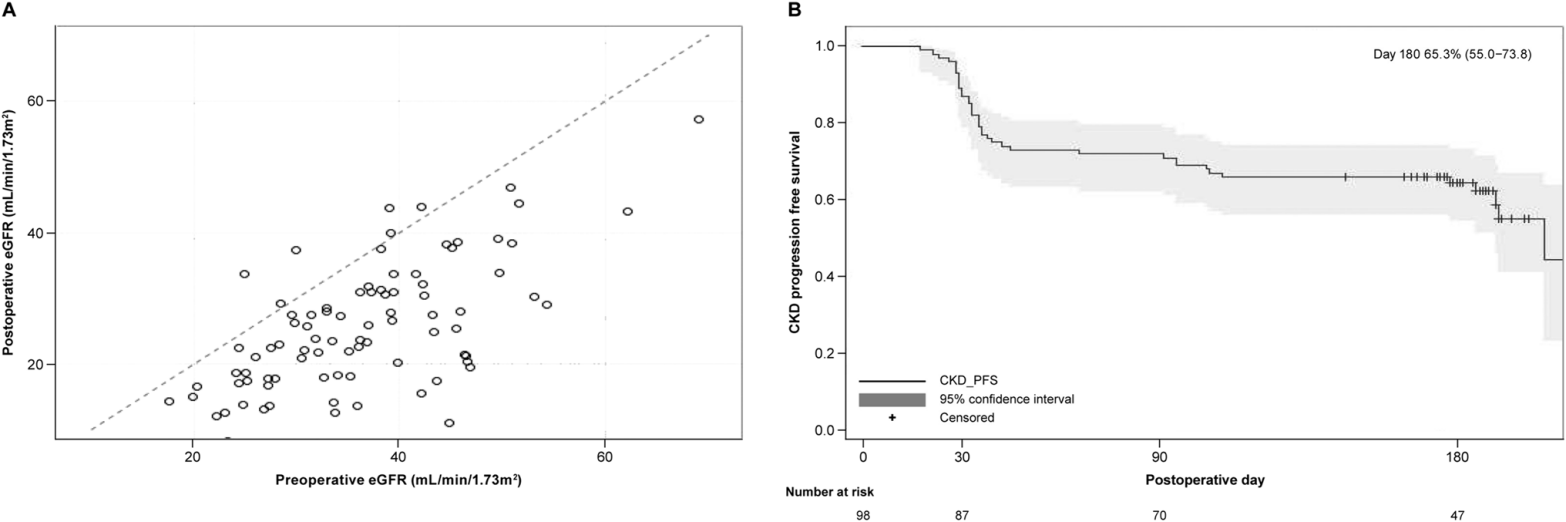
**A** The distribution of preoperative and postoperative (POD180) split eGFR (mL/min 1.73 m^2^) of operated kidneys. **B** Kaplan–Meier curve of CKD progression-free survival in RAPN. CKD progression is defined as the advancement of CKD by at least one stage in the CKD classification. The gray-shaded area represents the 95% CI

Our analysis revealed a mean ratio of split eGFR at POD180 for the baseline was 0.707 (95% CI 0.66–0.751) Fig. 3A) showed the relation between pre- and post-operative split eGFR. The Kaplan–Meier curve highlighted a significant drop in renal function immediately after surgery, which plateaued after that. By POD 180, 63.5% of patients (95% CI 55.0–73.8) showed no CKD progression (Fig. 3B).

### Safety assessment

Overall, 12 adverse events were identified during the perioperative period, which is within POD30. Among these, five events were related to surgery: grade < 3 included one case of hypothermia and one case of pneumonia, while grade ≥ 3 included two cases of urinary fistula and one case of intraoperative bleeding. From POD30 to POD180, three neoplastic lesions occurred, including one case each of recurrent lung cancer, bladder cancer, and colon carcinoid, which were considered unrelated to the current study.

### Factors influencing postoperative renal function

According to a multivariate analysis, the selected model (Table 3) identified several perioperative factors associated with the renal function of the operated kidney (percentage of postoperative eGFR against preoperative eGFR).

**Table 3:** Factors affecting postoperative renal function (percentage postoperative split eGFR against preoperative split eGFR)

Factors	Variables	Univariable analysis			Multivariable analysis		
		Estimate (95% CI)		P-value	Estimate (95%CI)		P-value
Age	Age (year)	0.09	(− 0.24, 0.43)	0.580	0.01	(− 0.29, 0.32)	0.940
Sex	Sex (ref = male)						
	Female	− 1.46	(− 10.56, 7.64)	0.751	− 3.91	(− 11.67, 3.85)	0.319
Preoperative renal function	eGFR (pre)	− 0.2	(− 0.46, 0.05)	0.117			
	eGFR (pre) (operated kidney)	− 0.2	(− 0.65, 0.25)	0.381	− 0.21	(− 0.62, 0.21)	0.325
Operative complexity	PADUA score (/score)	− 3.62	(− 6.76, − 0.48)	0.025			
	PADUA score (ref = moderate)						
	High	− 8.95	(− 17.92, 0.02)	0.054			
	R.E.N.A.L. score (/score)	− 6.56	(− 9.82, − 3.29)	< 0.001			
	R.E.N.A.L. score (ref = low)						
	Moderate	− 15.24	(− 33.74, 3.25)	0.105	− 11.54	(− 28.66, 5.58)	0.184
	High	− 24.13	(− 42.88, − 5.38)	0.012	− 12.41	(− 30.05, 5.22)	0.165
Surgical time	Surgical time (10 min)	− 1.34	(− 2.04, − 0.63)	< 0.001	− 0.85	(− 1.58, − 0.11)	0.024
Warm ischemic time	Warm ischemic time (/min)	− 0.8	(− 1.44, − 0.15)	0.017			
	Warm ischemic time (ref = under 25)						
	Over 25	− 16.78	(− 28.99, − 4.57)	0.008	− 11.12	(− 22.34, 0.1)	0.052
Resected weight	Resected weight	− 0.74	(− 1.01, − 0.46)	< 0.001	− 0.52	(− 0.84, − 0.21)	0.002

The estimate is the effect for a percentage of post-/pre-split renal function of operated kidney

The surgical time, weight of the resected renal mass, and WIT were all predictors of postoperative renal function. Specifically, for every additional 10 min of surgical time, renal function declined by 0.85% (95% CI  − 1.58 to − 0.11), for each gram of resected renal weight, it decreased by 0.52% (95% CI  − 0.84 to − 0.32), and for WIT exceeding 25 min, the reduction was 11.23% (95% CI  − 22.34 to − 0.12). Among patients with a WIT exceeding 25 min., only one exhibited a postoperative split renal function above 80% against preoperative function (Supporting Figure S1).

## Discussion

Our study of RAPN for completely endophytic renal tumors has yielded significant results, especially in terms of the two primary endpoints (WIT and PSM rate). The observed WIT of 20.3 min (99% CI 18.3–22.3) not only significantly surpassed the historical values associated with cLPN but also remained well below the critical 25-min benchmark crucial for preserved postoperative renal function. The achievement of a 0% in PSM (99% CI 0–5.3%) reflects the oncological efficacy of RAPN, particularly in managing the complexities of completely endophytic tumors. Successfully meeting both primary endpoints clearly demonstrated functional and oncological efficacy of RAPN.

Numerous studies have compared RAPN with cLPN [6–8, 16–18], and although there is general agreement that RAPN offers superior outcomes, the specific variables where RAPN outperforms cLPN vary between studies. Even among these reports, few are focusing on complex tumors. Overall, the findings contribute to the consensus that RAPN is becoming the standard approach, including complex tumors such as completely endophytic tumors.

There has been a recent trend toward individualized evaluations for surgically challenging tumors. Recent reports have highlighted the shorter WIT in RAPN compared with cLPN for renal hilar tumors, as comparably challenging as completely endophytic tumors [9, 10]. However, studies focusing on completely endophytic tumors, which present even greater surgical challenges, are less common. Observational studies [19–21] indicate a WIT of 17–27 min in RAPN for these tumors, which is consistent with our findings. As noted in these study, RAPN, which can be operated in 3D, may be superior to cLPN in highly difficult surgical procedures. This lack of direct comparisons between cLPN and RAPN for completely endophytic tumors underscores the unique contribution and importance of our study. Furthermore, our results highlight the critical role of RAPN in renal function preservation, with WIT averages consistently below the crucial 25-min threshold.

The concept of “trifecta” in partial nephrectomy, initially introduced by Hung et al*.* [22], encompasses three key outcomes: negative cancer margins, minimal renal functional decrease, and absence of urological complications. It is noteworthy that various papers have modified the latter two criteria. Our study tailored these criteria to evaluate midterm outcomes, specifically defining them as maintaining an eGFR of 90% or above the preoperative level at POD 180 and the absence of significant complications within POD 180.

The trifecta achievement rate of 47.96% (95% CI 38.33–57.74) in our study is notable despite being lower than previously reported rates (61.7–84.1%) [22–24]. Considering our 81.6% achievement rate of WIT ≤ 25 min, the difference in trifecta achievement rate between present and previous studies is likely due to differing criteria for renal function. While they set WIT, we based on renal function at POD 180. In studies that expanded the trifecta criteria to include eGFR evaluations several months postsurgery, the reported attainment rates ranged from 54.6 to 67%, slightly higher than that in the present study [24, 25]. This discrepancy suggests that factors beyond early functional success may influence the preservation of midterm renal function. A direct evaluation is difficult to perform because the viewpoints of renal function differ for each paper; however, it seems that 3.2%–50% of patients may have decreased renal function after RAPN [18, 24–26].

Our study contributes to understanding the factors influencing postoperative renal function in RAPN. Post-PN renal function had been evaluated by several outcomes such as acute kidney injury [27], split renal functions [28], trifecta or pentafecta [23, 25], or progress of CKD grade [4]. In this study, we evaluated renal function using split renal function, following the previous report [10]. Although preoperative comorbidities such as age, body mass index, and comorbidities are known to impact renal outcomes [29], our analysis focused on surgical factors. We found that the resection weight, WIT, and overall surgical time significantly influenced the postoperative split renal function of operated kidney, Reported surgical factors include the R.E.N.A.L. score, pentafacta, WIT, and bleeding [4, 21, 30]. WIT is often considered to be around 25 min; however, there are also reports that it can have an impact in min [4, 5]. Our findings indicate that although patients with WIT under 25 min exhibit a mix of preserved and decreased renal function, those with WIT over 25 min consistently show decreased renal function, with a few exceptions, suggesting that 25 min is a reasonable threshold, as cases preserving renal function beyond this duration were rare. Several reports have indicated that the R.E.N.A.L. score impact renal function after PN, and in present study, inclusion criteria specifically limited the “R” and “E” factors of the R.E.N.A.L. score. Consequently, these did not exhibit the same impact on renal function as reported previously. In this cohort, the category labeled as “Low risk” within “E” factor was not represented. This absence of the “low risk” group in “E” factor might account for this result. There have been reports suggesting an association with tumor diameter. Resection weight, often dictated by tumor size, was also correlated with achieving negative surgical margins. It is critical to balance the extent of resection with the need to preserve renal function while avoiding excessive healthy renal parenchymal removal. These factors may indicate that enhancing surgical skills and techniques can improve patient outcomes.

Looking forward, while this report has focused on midterm renal function and surgical efficacy, our ongoing research will extend the follow-up period to five years. This long-term follow-up will provide a more comprehensive picture of RAPN outcomes, including sustained renal function and recurrence rates. The forthcoming data will be crucial in further establishing the long-term benefits and potential limitations of RAPN, thereby contributing to the continuous evolution of surgical strategies in renal cancer treatment.

A major limitation of our study is the comparison with historical cLPN data, which may affect the comparative validity. Nevertheless, the use of 99% CIs helps mitigate some uncertainties in our analysis. In addition, renal function after PN has been evaluated using various definitions, and influencing factors may also differ depending on the definition employed. The constant evaluation of renal function will become necessary in the future.

## Conclusion

In conclusion, RAPN shows clear superiority over cLPN (especially in terms of WIT) and proves effective in performing complex surgical procedures such as those performed for completely endophytic tumors, as evidenced by favorable surgical margin rates. The key predictors of midterm renal function in our study were the total surgical time, WIT, and resection weight.

## Supplementary Information

Below is the link to the electronic supplementary material.

Supplementary file1 (DOCX 67 KB)

Supplementary file2 Figure S1***: Relationship between WIT and the ratio of the postoperative/preoperative split eGFR (TIF 349 KB)

## Data Availability

The data sets generated during and/or analyzed during the current study are available from the corresponding author on reasonable request.
