# Supplementary material for: Efficacy of robot-assisted partial nephrectomy compared to conventional laparoscopic partial nephrectomy for completely endophytic renal tumor: a multicenter, prospective study
Source: Int J Clin Oncol. 2024 Aug 7;29(10):1548–56. doi: 10.1007/s10147-024-02599-9 (PMC11420261; doi:10.1007/s10147-024-02599-9)
Supplement: Supplementary file 1 — Supplementary file1 (DOCX 67 KB) [file 10147_2024_2599_MOESM1_ESM.docx]

Supplementary Table S1. Patients characteristics of historical control

|  | **Control studies** | | | |
| --- | --- | --- | --- | --- |
| **Baseline characteristics** | **Chung BL *et al.*** | **Nadu A *et al.*** | **Di Pierro GB *et al.*** | **Fan G *et al.*** |
| Patients (*n*) | 55 | 41 | 11 | 5 |
| Women, *n* (%) | NA | NA | NA | 2 |
| Mean age, years (range) | NA | 59.4 (SD:5.3) | 45.3 (38.2–64.1) | 55 (50–61) |
| Mean BMI, kg/m^2^(range) | NA | NA | 24.3 (22.5–2 6.5) | 22.4 (19.9–24.1) |
| ASA–PS category, *n(%)*  1  2  3  NA | NA | NA | 6 (55) 5 (45) 0 0 | 2 (40.0) 3 (60.0) 0 0 |
| Mean eGFR, ml/min/1.73 (range) | NA | 84.14 (SD:27.1) | 78 (67–89) | NA |
| Mean tumor diameter, cm (range) | 2.3 (1.0–4.5) | 2.6 (SD:0.8) | 1.6 (1.2–2.0) | NA |
| Perioperative data |  |  |  |  |
| Mean surgical time, min (range) | NA | 118.9 (SD:24) | 140 (110–200) | 162.6 (105–221) |
| Mean estimated blood loss, mL (range) | 236 (25–1000) | 279.5(SD:NA) | 270 (100–750) | 81 (50–125) |
| Mean WIT, min (range) | 29.9 (7–50) | 22.6 (SD:13.8) | 24 (19–32) | 23.4 (18–27) |
| Perioperative complications |  |  |  |  |
| Total (*n*) | NA | 10 | 2 | 0 |
| Clavien grade I/II (*n*) | NA | 4 | 1 | 0 |
| Clavien grade III/IV (*n*) | NA | 6 | 1 | 0 |
