# Supplementary figures and images for: Efficacy of robot-assisted partial nephrectomy compared to conventional laparoscopic partial nephrectomy for completely endophytic renal tumor: a multicenter, prospective study
Source: Int J Clin Oncol. 2024 Aug 7;29(10):1548–56. doi: 10.1007/s10147-024-02599-9 (PMC11420261; doi:10.1007/s10147-024-02599-9)

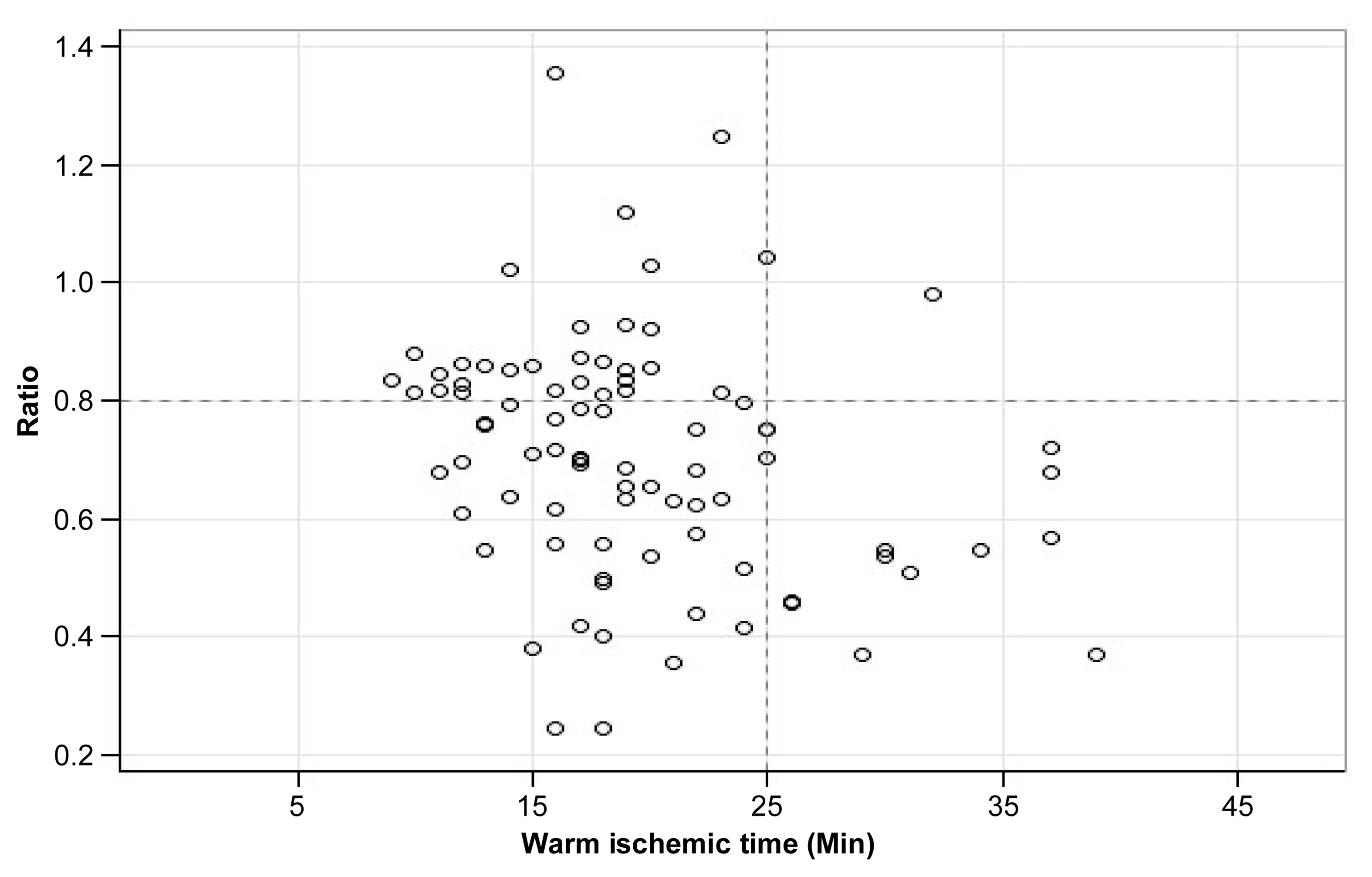

Supplement: Supplementary file 2 — Supplementary file2 Figure S1***: Relationship between WIT and the ratio of the postoperative/preoperative split eGFR (TIF 349 KB) [file 10147_2024_2599_MOESM2_ESM.tif]
